# Hormonal Decline and Neurotransmitter Dysregulation in Menopause: Pathways of Psychiatric Vulnerability

**DOI:** 10.1192/j.eurpsy.2026.11951

**Published:** 2026-07-31

**Authors:** G. J. Cowen, D. Valle, T. Waldmann, A. Gasparova, L. De Faria, B. R. Carr

**Affiliations:** 1 School of Medicine, Wayne State University, Detroit, MI; 2 College of Medicine; 3 Department of Psychiatry, University of Florida, Gainesville, FL, United States

## Abstract

**Introduction:**

Menopause entails marked hormonal fluctuations, yet the neurobiological mechanisms linking these shifts to psychiatric vulnerability remain underintegrated in psychiatric models. Declines in estrogen and in neurosteroids such as allopregnanolone and 5α–androstane–3α,17β-diol (3α–ADIOL) have been directly implicated in neurotransmitter dysregulation, contributing to heightened risk for mood and anxiety disorders in perimenopausal women.

**Objectives:**

To synthesize current evidence on neuroendocrine and neurotransmitter mechanisms of psychiatric vulnerability during menopause, focusing on estradiol–serotonin interactions and neurosteroid–GABA-A modulation.

**Methods:**

We conducted a narrative review of mechanistic and translational studies examining the impact of declining estrogen and neurosteroid concentrations on monoamine oxidase activity, serotonergic bioavailability, and GABA-A receptor function during menopause.

**Results:**

Estradiol inhibits monoamine oxidase A and B, enhancing synaptic serotonin; perimenopausal decline disrupts this regulation, contributing to mood and anxiety symptoms. Similarly, reduced allopregnanolone levels after menopause attenuate positive allosteric modulation of GABA-A receptors, weakening inhibitory signaling and predisposing to hyperarousal, anxiety, and sleep disturbance. Furthermore, postmenopausal women exhibit lower androgen levels relative to premenopausal women. The androgenic neurosteroid 3α–ADIOL functions as a positive allosteric modulator of GABA-A receptors, and reduced concentrations have been inversely correlated with anxiety expression. Collectively, these findings support a model in which menopausal hormonal decline disrupts homeostatic serotonergic and GABAergic regulation, thereby amplifying vulnerability to psychiatric symptoms.

**Conclusions:**

Hormonal decline during menopause disrupts serotonergic and GABAergic pathways via estrogen loss and diminished neurosteroid modulation. These mechanistic insights underscore menopause as a critical window for targeted intervention, supporting exploration of estrogen replacement and novel GABAergic modulators to mitigate psychiatric risk.

**Disclosure of Interest:**

None Declared

